# Dedifferentiation of Functional Brain Activation Associated With Greater Visual Discrimination Accuracy in Middle-Aged and Older Adults

**DOI:** 10.3389/fnagi.2021.651284

**Published:** 2021-07-12

**Authors:** Talia R. Seider, Eric C. Porges, Adam J. Woods, Ronald A. Cohen

**Affiliations:** Center for Cognitive Aging and Memory, Clinical Translational Research Program, Department of Clinical and Health Psychology, University of Florida, Gainesville, FL, United States

**Keywords:** dedifferentiation, compensation, visual discrimination, fMRI, age, PASA, visual assessment battery

## Abstract

Neural dedifferentiation refers to an age-related phenomenon whereby brain functions that are localized to specific, distinct, and differentiated brain areas in young adults become less so as people reach more advanced age. Older adults tend to exhibit greater spread of cortical activation on fMRI during cognitive processing compared to younger adults, with evidence that this occurs during visuoperceptual processing. Some age-related functional changes are considered compensatory, but whether dedifferentiation is compensatory is not clearly understood. The current study assessed dedifferentiation and visual discrimination performance during simultaneous match-to-sample tasks from the Visual Assessment Battery (VAB) among 40 healthy middle-aged and older adults using fMRI. Task-relevant regions of interest (ROIs) were created in the dorsal stream for discrimination of spatial location, the ventral stream for shape, and an area encompassing V5 for velocity. Dedifferentiation, or less specificity in functional activation, was associated with greater discrimination accuracy and more years of education. Secondary analyses showed that reduced functional activation in task-relevant ROIs was associated with faster discrimination speed. Age was unassociated with functional activation. Results suggest that dedifferentiation is compensatory. Lack of age effects suggest that other factors beyond age, such as cognitive or brain reserve, may better predict performance when considering cognitive skills that are relatively stable as adults age, such as visual discrimination.

## Introduction

Advanced age is associated with loss of brain volume, particularly in frontal regions, as well as a reduction in white matter integrity (Raz et al., 2005; Madden et al., 2012). Functional changes have also been reported, such as neural dedifferentiation. This refers to an age-related phenomenon whereby brain functions that are localized to specific, distinct, and differentiated brain areas in young adults become less so as people reach more advanced age. For example, functional activation patterns that are more clearly differentiated in younger adults (e.g., processing of faces generally occurs using the lingual and fusiform gyri, and processing of places preferentially recruits the parahippocampal place area) are less differentiated in older adults (Grady et al., 1992; Grady et al., 1994; Park et al., 2004; Voss et al., 2008). Not only do older adults activate more “face” regions when viewing places and more “place” regions when viewing faces (compared to younger adults), but there is increased functional activation in each of those areas in response to a greater variety of visual stimuli in older adults than in younger adults. Thus, functional activity in the older brain is less specialized.

Dedifferentiation may help older adults compensate for brain changes (Reuter-Lorenz and Cappell, 2008). Consistent with this idea is evidence from stroke patients, who recruit brain areas adjacent to the damaged tissue as well as areas analogous to the damaged tissue in the undamaged hemisphere when engaged in a task that would otherwise utilize the damaged region (Buckner et al., 1996; Cao et al., 1999; Logan et al., 2002). Another piece of evidence consistent with the compensation hypothesis came from a study of transcranial magnetic stimulation (TMS), which can reduce function in the area to which it is applied. In a visual memory task on which older and younger adults performed comparably at baseline, TMS only interfered with performance in younger adults when applied to the right hemisphere, but it impacted performance in older adults regardless of which hemisphere received the TMS. This suggested that performance in older adults is substantially more dependent on both hemispheres to perform as well as younger adults (Rossi et al., 2004). However, dedifferentiation has also been associated with reductions in performance in visual tasks (Voss et al., 2008). Overall, there is limited research examining dedifferentiation as it relates to performance in older adults to determine whether this activation change is, in fact, compensatory.

In a previous study, we demonstrated that greater age was associated with greater anterior functional activation, especially in the bilateral middle frontal gyri, during visual discrimination among cognitively healthy middle-aged and older adults (Seider et al., 2020). There were age-related declines in processing speed but not accuracy. Yet, it remains to be seen how visual discrimination performance and neural activity were related. The current study examined whether age-associated dedifferentiation existed during visual discrimination tasks, and whether functional activation was related to performance. Three discrimination tasks were derived from the Visual Assessment Battery (VAB) (Swearer and Kane, 1996), which employs a simultaneous match-to-sample paradigm in which tasks differ based on whether perceptual discrimination of shape, location, or velocity is required, with three levels of difficulty for each task. VAB tasks were selected to activate three different processing streams: the superior “where” pathway, the inferior “what” pathway, and a motion-sensitive visual region containing V5/MT (Seider et al., 2020). Measures of accuracy and response time (RT) were obtained and analyzed relative to the specificity of neural activation on fMRI in task-associated regions of interest (ROIs) to determine whether dedifferentiation occurred as a function of age and whether it corresponded with perceptual performance.

There are potential functional implications based on whether there is highly specific correspondence between performance and functional activation in task-specific ROIs. If greater spread of activation, to cortical areas that extend beyond the primary task-associated ROI, is associated with stronger discrimination performance, it would provide evidence for dedifferentiation being a beneficial compensatory response. If the opposite relationship were found, with greater spread of activation associated with poorer performance, then the results would suggest that dedifferentiation is not beneficial or indicative of effective compensation. The authors hypothesized that greater dedifferentiation would be associated with better performance, suggesting that it is compensatory. In studies comparing older to younger adults (Grady et al., 1992; Park et al., 2004; Voss et al., 2008), dedifferentiation increases with age, but given the relatively restricted age range of the study sample, large age effects were not expected.

## Materials and Methods

### Participants

Forty healthy adults ages 51–91 were recruited from a larger dataset of community-dwelling adults enrolled in the Active Brain study, a University of Florida (UF) neuroimaging study aimed at investigating brain activity in healthy older adults. The sample size was chosen based on study group size in similar fMRI research showing significant findings (Ryan et al., 2012). Inclusion and exclusion criteria are listed in Table 1. Participant characteristics are listed in Table 2. Ten participants were gathered for age decades 50–59, 60–69, 70–79, and 80+. They were college educated, on average, 55% female, and predominantly Caucasian (*N* = 38/40). Participants were free from pre-existing dementia or other neurological disease, major psychiatric disorders, history of head injury with loss of consciousness greater than 15 min, and major visual impairments. Exclusionary criteria were verified via medical history questionnaires, interview, and testing of cognitive and visual function. All participants had or were corrected to 20/40 vision or better. Participants were also free of conditions or implants for which MRI is contraindicated, including claustrophobia. Participants were given information about the study and, if they were interested in participating, provided verbal and written informed consent to participate.

**TABLE 1:** Inclusion and exclusion criteria.

**Inclusion**	**Exclusion**
Age 50+	Neurological disease including dementia
Normal or corrected-to-normal vision	Major psychiatric disorder
MRI compatible	History of head injury (LOC > 15 min)
Enrolled in Active Brain study	Diseases heavily affecting vision

**TABLE 2:** Participant characteristics.

	**Mean (SD)**	**Range**
Age (years)	69.9 (11.7)	51–91
Education (years)	15.8 (2.5)	12–20
MoCA	26.6 (2.2)	21–30
% Male	45	
% Caucasian	95	

*SD, Standard deviation.*

### Stimuli

**FIGURE 1:**
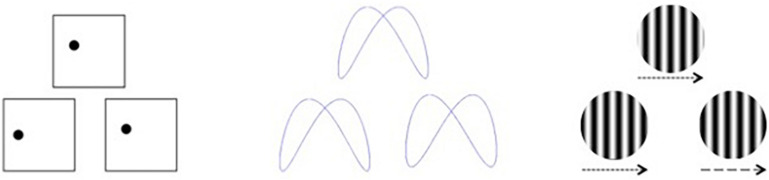
Visual stimuli for discrimination based on spatial location **(left)**, shape **(middle)**, and velocity **(right)**. Arrows indicate movement velocity for the purposes of this figure and were not included in the actual test stimuli.

Visual stimuli were created using E-Prime 2.0 software based on a subset of those used by Swearer and Kane (1996), which we described previously (Seider et al., 2020). Participants were presented with simultaneous matching paradigms requiring perceptual judgments of either spatial location, shape, or velocity (Figure 1). For each task, three stimuli were presented, with two located side-by-side below the horizontal midline of the display and one located above the horizontal midline and centered on the vertical midline. The upper stimulus (target) was identical to one of the lower (sample) stimuli, and participants were asked to indicate which of the sample items was identical to the target via button press. The tasks varied over three levels of difficulty (easy, medium, and hard). For Location, the task was to decide which sample box below had a dot in the same location as the target box above. Boxes were 6.65-cm squares, and difficulty was based on how far the dot in the sample item was located from the dot in the target item, either 1.27, 0.79, or 0.48 cm from the target location. For Shape, the task was to decide which sample design below was identical to the target design above. Difficulty was based on judgment RTs, which were highly correlated to difficulty ratings, obtained during a pilot study. For Velocity, the task was to decide which sample set of lines below were moving at the same speed as the target set of lines above. Difficulty was based on the speed differences between the sample and target items, which differed by a factor of the just noticeable difference (JND) for that velocity (either nine, six, or three JNDs).

### Procedure

fMRI scanning took place at the UF McKnight Brain Institute (MBI) Advanced Magnetic Resonance Imaging and Spectroscopy (AMRIS) facility and lasted approximately 1 h. Prior to entering the scanner, participants were screened using the MRI safety screening form for research participants. They were given a clear and detailed explanation regarding the MR procedure and trained on the experimental paradigm. Those requiring glasses received MRI-compatible lenses to correct vision, with glasses prescription measured by lensometer. During scanning, participants were positioned supine, with cushioning surrounding their heads to reduce movement artifact. Structural imaging was acquired first, during which participants were instructed to be still and relax. This was followed by functional imaging, during which they completed the cognitive task.

To ensure that each participant understood the nature of the task and were comfortable performing it in the scanner, they completed a practice block in the MRI with nine trials, consisting of one trial for each of the three levels of difficulty within each of the three tasks. All stimuli were projected onto an LCD screen (BOLDScreen 32, Cambridge Research Systems) behind the scanner and were viewed via a mirror positioned above participants’ heads. Participants were instructed to indicate which of the two lower sample stimuli was identical to the upper target stimulus (Figure 1) by pressing one of two buttons on a response box, using their right middle and index fingers. They were asked to respond as quickly and as accurately as possible, but not to sacrifice accuracy for speed. They were given 3 s to respond. Accuracy and RT were recorded. Prior to each stimulus, a centrally located fixation cross was presented to direct attention the center of the screen.

### fMRI Data Acquisition

fMRI data was acquired on a 3T Philips Achieva scanner (Philips Healthcare, Best, Netherlands) at the UF-MBI AMRIS facility using a 32-channel sensitivity encoding (SENSE) head coil. Anatomical images were acquired using a whole-brain high-resolution 3D MP-RAGE sequence: 176 1 mm thick sagittal slices, TR = 7 ms, TE = 3.2 mc, FA = 8°, matrix = 256 × 256, FOV = 256 mm. Functional images were acquired in the axial-oblique plane using a Phillips 3T scanner: 36 slices, TR = 2 s, TE = 30 ms, FA = 80°, FOV = 224 mm × 224 mm, acquisition matrix 64 × 64, isotropic voxels of 3.5 × 3.5 × 3.5 mm^3^. Prior to the collection of each run, a number of dummy scans were collected and discarded to allow for the signal to stabilize.

**FIGURE 2:**
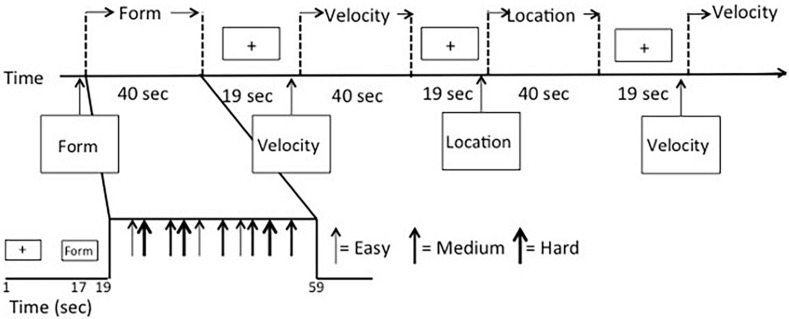
fMRI mixed block/event-related design.

Data were collected over three functional scans using a mixed block/event-related design (Figure 2) to allow for analysis in either a block- or event-related fashion. Scans began with a 17-s fixation cross presented in the middle of the screen, followed by a 2-s instruction screen that informed participants as to the type of discrimination task to follow (Location, Shape, or Velocity). Following the instruction screen, a block of seven stimuli was presented. Each stimulus displaying a simultaneous match-to-sample task was on the screen for 3 s. Responses were recorded during either this time or the fixation time that immediately followed. Fixation time between stimulus events (inter-stimulus intervals) was randomly jittered with a minimum of 1000 ms and an average of 2500 ms to increase the temporal resolution of the estimated hemodynamic response. Blocks of activity were separated by 17-s fixation periods. Another fixation period of 17 s was presented at the end of the block. The first two scan runs had 14 full blocks and ran a total time of 824 s, while the third scan run had 14 blocks, three of which only had five stimuli instead of seven, and it ran for a total of 792 s. Task types were evenly divided between blocks, so there were 14 blocks for each VAB task (Location, Shape, and Velocity). Scan order, block order, and stimulus presentation within the block were randomized. Each block contained a mix of task type and difficulty levels. There were 288 stimuli total, 32 stimuli for each of the nine conditions (three tasks with three levels of difficulty each).

### fMRI Data Analysis

fMRI data pre-processing and analyses were performed using Statistical Parametric Mapping (SPM12) software^1^. Slice timing correction was used to correct differences in image acquisition time between slices using the middle slice as a reference. Motion correction in functional data was addressed via SPM realign, using a least squares approach and a six-parameter (rigid-body) spatial transformation; a two-pass procedure registered the images to the mean of the images after the first realignment. Realignment parameters were used to exclude runs that had substantial motion artifacts based on researcher consensus, and motion regressors were included in the first level to account for movement. Functional data were co-registered to the subject’s anatomical image using a rigid-body model. Anatomical data were segmented, bias-corrected, and spatially normalized to a standard Montreal Neurological Institute (MNI) template. These transformations were applied to the functional data, normalizing them to MNI space. Data were then spatially smoothed with a Gaussian kernel of 8 mm to suppress noise and effects due to residual differences in functional and structural anatomy during inter-subject averaging.

Scan runs with less than 68% accuracy (i.e., performances approaching chance) were excluded to avoid measuring functional activity during poor effort or attention. One participant was excluded entirely (not included in the *N* = 40 described above), three participants had one scan run excluded, and two had two runs excluded. One participant had only two scan runs collected due to mechanical difficulties that stopped the MRI during the third scan. Data was analyzed in an event-related fashion to allow for examination of each difficulty level.

**FIGURE 3:**
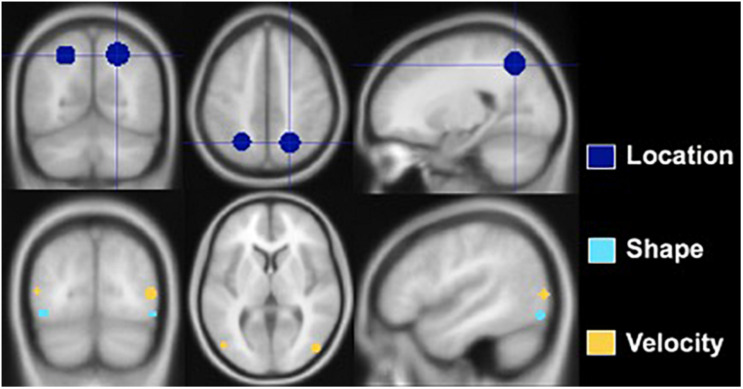
Regions of interest constructed for Location, Shape, and Velocity. The Location ROI is situated in the posterior parietal lobe, the Shape ROI in the lateral occipital, and the Velocity ROI at the junction of the occipital, temporal, and parietal lobes.

Regions of interest were created using the MarsBaR toolbox for SPM 8, and analyses were conducted in a MATLAB environment (Math-works, Natick, MA, United States). Spherical ROIs were designed based on prior research. Figure 3 depicts the ROIs, and Table 3 lists the MNI coordinates on which they are centered and radii. The Location ROI was designed to be in the dorsal processing stream based on established findings (Mishkin et al., 1983; Livingstone and Hubel, 1988). MNI coordinates for the posterior parietal cortex (PPC) were extracted from a study that required participants to judge the distance between a dot and a line across two panels and respond as to whether the distances were the same or different (Zachariou et al., 2013). PPC activation compared to a control task (slide matching without distance estimation) centered at MNI coordinates −25 −60 49 with a radius of 9.2 mm for the left hemisphere and 22 −61 50 with a radius of 10.6 mm for the right hemisphere. Shape tasks consistently activate the lateral occipital region (Jiang et al., 2007; Pitcher et al., 2009; Silvanto et al., 2010; Zachariou et al., 2013); thus, the Shape ROI was located there. Coordinates were extracted from a study that subtracted activation during viewing of scrambled car images from activation during viewing of car images (Jiang et al., 2007). The left hemisphere ROI was centered at MNI coordinates −43 −78 −10 with a radius of 4 mm, and the right hemisphere ROI was centered at MNI coordinates 48 −75 −11 with a radius of 4 mm. The Velocity ROI was designed to be in V5/MT based on established findings (Zeki et al., 1991; Orban et al., 2003), but the coordinates and radii for the spherical ROIs created were based on a study of retinotopic organization of that area (Kolster et al., 2010). The left hemisphere ROI was centered at −48 −75 8 and had a 4-mm radius, and the right hemisphere ROI was centered at 22 −61 50 and had a 4.6-mm radius. The larger right vs. left hemisphere ROI was consistent with our findings that the velocity task uniquely elicited more right than left hemisphere activity (Seider et al., 2020).

**TABLE 3:** MNI coordinates of the center of regions of interest in the left and right hemispheres and radii in millimeters.

**ROI**	**Left hemisphere**				**Right hemisphere**			
	*X*	*Y*	*Z*	Radius	*X*	*Y*	*Z*	Radius
Location	−25	−60	49	9.2 mm	22	−61	50	10.6 mm
Shape	−43	−78	−10	4 mm	22	−61	50	4.6 mm
Velocity	−48	−75	8	4 mm	46	−78	6	4.6 mm

*Montreal Neurological Institute (MNI) coordinates *x* (left-right) *y* (anterior–posterior) *z* (inferior-superior). ROI, region of interest.*

### Statistical Analysis

**FIGURE 4:**
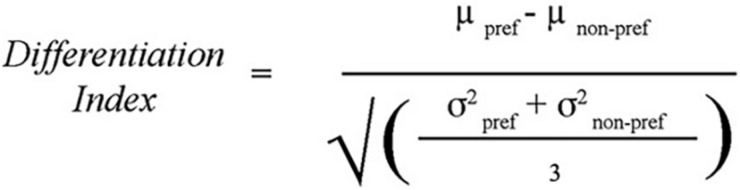
Differentiation Index: used to calculate the degree to which task-associated ROIs were uniquely recruited for the preferred task compared to the non-preferred tasks. For example, the differentiation index for the Location task was computed as the percent signal change within the Location ROI during the Location task minus the average percent signal change within the Location ROI during the Shape and Velocity tasks, divided by the average standard deviation of percent signal change to all three tasks within the Location ROI. Method from Voss et al. (2008).

To test whether there was age-related dedifferentiation, a differentiation index was created as a measure of discriminability. The measure is calculated by subtracting the mean functional activation for the non-preferred tasks in the task-associated ROI from that of the preferred task in that ROI and dividing by a measure of the mean variability of activation for all tasks within that ROI. This method has been described by Voss et al. (2008), and the formula used was reproduced from their paper (Figure 4). In effect, the differentiation index measures the degree to which an ROI is differentially activated during the task that is theoretically specialized to that region vs. other similar tasks. A bivariate correlation compared age with the differentiation indices for each of the three tasks (Location, Shape, and Velocity).

Behavioral performance was based on median RT for a task, used instead of mean because of the tendency of RTs to be positively skewed (Van Zandt, 2002), as well as accuracy. Composite performance scores were created by averaging the mean of the *z*-score for accuracy and the reversed *z*-score for median RT for each of three visual discrimination tasks, difficulty levels collapsed. Difficulty levels were collapsed because the parameters underlying difficulty differed for each task, so comparing easy, medium, and hard items across tasks was not possible. To determine whether dedifferentiation was associated with performance, composite performance scores for each task were correlated with the degree to which associated ROIs were differentially activated for those preferred tasks (differentiation index). Additionally, correlation analyses compared performance composite scores for each task (difficulty levels collapsed) to percent signal change in its task-specific ROI.

## Results

**FIGURE 5:**
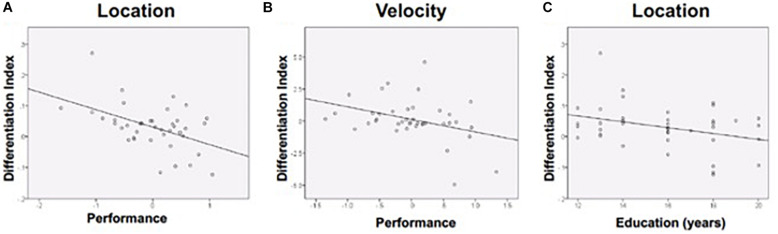
Differentiation was negatively associated with **(A)** Location performance, **(B)** Velocity performance, and **(C)** Education (during location discrimination only). Performance is a composite *z*-score of response time and accuracy.

The differentiation index reflects the specificity of functional activation on each task (i.e., larger value indicates greater functional specificity, or differentiation, while smaller values indicate greater *de*differentiation). The differentiation index ranged from close to 0, which indicates complete dedifferentiation, to higher values that suggest greater activation in the preferred vs. non-preferred ROI. Age was unrelated to differentiation. Discrimination performance was negatively associated with magnitude of the differentiation index for the Location and Velocity tasks. Stronger performance on the Location task was associated with a smaller differentiation index (i.e., greater *de*differentiation: *r* = −0.476, *p* = 0.002) (Figure 5A). Stronger performance on the Velocity task was also associated with a smaller differentiation index (*r* = −0.365, *p* = 0.021) (Figure 5B). For Shape discrimination, the relationship between the discrimination index and performance was not statistically significant (*r* = −0.219, *p* = 0.175). Yet, the small effect suggests a similar negative relationship between performance and the extent of functional activation differentiation across ROIs.

Follow-up analyses were conducted in attempts to further explain our findings. Given that education is widely used as a proxy measure of cognitive reserve, which may impact how the brain compensates for age-related changes (Stern, 2009), follow-up correlational analyses compared education to the differentiation index. More years of education was associated with lower differentiation (more *de*differentiation) for Location discrimination (Figure 5C), consistent with prior findings showing that dedifferentiation may be adaptive.

Follow-up analyses also revealed that RT and accuracy performance differed in their relationship to discrimination indices. Smaller differentiation index values (greater indices of *de*differentiation) were associated with greater Location accuracy (*r* = −0.426, *p* = 0.006) and greater Velocity accuracy (*r* = −0.322, *p* = 0.043). The differentiation index was not significantly associated with RT on any of the discrimination tasks. Thus, accuracy appeared to account for the relationship between better performance and greater dedifferentiation.

**FIGURE 6:**
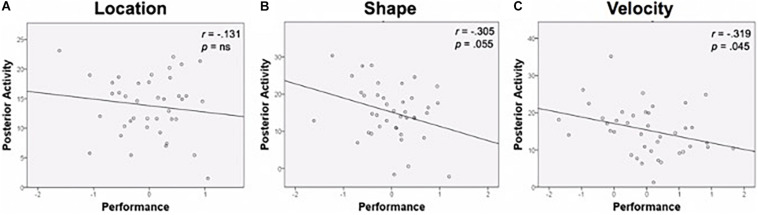
Performance and posterior activity. **(A)** Location task performance and activity in the Location ROI. **(B)** Shape task performance was negatively related to activity in the Shape ROI. **(C)** Velocity task performance was negatively related to activity in the Velocity ROI. Performance is a composite *z*-score of response time and accuracy. ROI, region of interest.

Functional activation in task-specific regions was then examined relative to age and performance. Age was unassociated with posterior activation in the specific ROIs, so it was not included as a covariate. Bivariate correlations were conducted between task performance composite scores and percent signal change in task-relevant ROIs. Results are displayed in Figure 6. Performance was negatively correlated with activation magnitude for Shape (*r* = −0.305, *p* = 0.055) and Velocity (*r* = −0.319, *p* = 0.045); greater activation was associated with worse performance. A negative association existed for Location as well, though this relationship did not approach significance (*r* = −0.131, *p* = 0.422).

When examining the individual contributions of RT and accuracy to the performance-activation relationship, RT appeared to be driving the relationship, as there were no significant associations between accuracy and posterior ROI recruitment for any of the tasks. In contrast, Velocity RT was positively correlated with the amplitude of activation in Velocity ROI (*r* = 0.355, *p* = 0.02); greater activation was associated with slower performances.

In sum, greater dedifferentiation of functional activation was associated with stronger perceptual discrimination performance, particularly greater discrimination accuracy, and more education. Reduced magnitude of activation in task-relevant ROIs was associated with better performance, specifically faster discrimination speed. Age was unassociated with activation patterns.

## Discussion

The overarching goal of this study was to achieve greater understanding of age-associated differences in the functional response of cortical areas that play essential roles in visual perception. We previously demonstrated cortical areas with greatest functional activation during match-to-sample visual discrimination varied as a function of the primary perceptual demands of the task (Seider et al., 2020). The tasks required discrimination of either location, shape, or velocity at three difficulty levels. Location discrimination produced primary activation in the dorsal visual processing stream, shape discrimination in the ventral stream, and velocity discrimination in a cortical area encompassing V5. Greater age was not associated with either increased or decreased activation in task-related ROIs. These earlier findings raised questions regarding the extent and value of functional activation in cortical areas extending beyond the primary task-related ROI. While shape, location and velocity discrimination elicited maximal activation in the hypothesized task-related ROIs, was there dedifferentiation, or increased activity in other cortical areas involved in visual perception, among older adults? If so, was dedifferentiation beneficial or detrimental to perceptual performance? Evidence that functional activation largely remained in the primary task-associated cortical areas with advanced age would support continued functional neuroanatomic differentiation across the lifespan, whereas evidence that functional activation also occurred in other cortical visual areas would suggest dedifferentiation of cortical response in older adults. Analysis of the relationship between performance and dedifferentiation would reveal evidence as to whether there were beneficial or detrimental effects of age-associated dedifferentiation. In the current analyses, a differentiation index, calculated as the specificity of activation in the primary task-related ROI relative to the other cortical ROIs during each type of perceptual process, was used to address these questions.

Though maximum functional activation was evident in the primary cortical task-related ROI, depending on whether the ROI was involved in shape, location, or velocity perception, the two other visual cortical areas tended to activate as well. Some participants demonstrated considerable differentiation, using brain regions that were specialized to a particular task far more than using other visual processing streams, while other participants exhibited dedifferentiation, with greater cortical distribution of activation across visual areas. While causality cannot be inferred from correlations, the direct comparison of magnitude of dedifferentiation to the quality of visual discrimination performance provided a means of determining whether functional recruitment of additional cortical areas of the visual system was beneficial or detrimental. In every analysis in which a significant correlation between functional activation and performance was observed, greater dedifferentiation was associated with stronger discrimination performance. In other words, activation beyond the core task-related cortical area was not indicative of inefficient or impaired performance. To the contrary, individuals with less specificity of activation primarily in the task-related cortical area (i.e., those with greater dedifferentiation) tended to perform better. There has been considerable debate over the past decade around the functional significance of the recruitment of cortical areas beyond the region known to have functional anatomic relevance for a cognitive process (Buckner et al., 1996; Cabeza et al., 2004, 2020; Davis et al., 2008; Clement and Belleville, 2010; Berlingeri et al., 2013; Burianova et al., 2013; Daselaar et al., 2015; Crowell et al., 2020). In the context of visual discrimination performance, the current findings support the possibility that dedifferentiation serves a compensatory function. That greater dedifferentiation during the Location task was also associated with higher education lends evidence to its compensatory nature, as those with higher education may have more neural reserve and thus recruit brain networks more efficiently compared to those with less reserve (Stern, 2009).

When processing speed and accuracy were considered separately, only discrimination accuracy was significantly associated with the differentiation index. Greater dedifferentiation was associated with better accuracy in performance, but not faster responding. Behavioral results showed age-related reductions in processing speed, but not accuracy, on the VAB (Swearer and Kane, 1996). This may be why differentiation was unrelated to age. However, when considering recruitment of task-specific ROIs, it was processing speed that was associated with recruitment, as reduced posterior recruitment was associated with faster speed. Could this be compensatory as well?

Typically, greater functional activation in primary sensory regions is considered healthier, which contrasts with the current findings. Faster RTs have been associated with greater, not less, occipital activation (Oguz et al., 2003; Mohamed et al., 2004; Adleman et al., 2016), and a meta-analysis indicated that for studies in which younger adults performed better than older adults, they had greater occipital activation bilaterally (Spreng et al., 2010). However, comparing brain activity between younger and older adults without directly measuring the association between the activity and performance may mask the relationship between functional activity and behavior. Brain activity changes as a function of age-related alterations in vasculature and gray and white matter structure. There is less signal-to-noise in an older brain, which may reduce the extent of activation, and there may be differences in the hemodynamic response between younger and older adults that do not reflect differences in neuronal functioning. As such, studies must consider interaction effects between age and performance (Samanez-Larkin and D’Esposito, 2008) and must compare activation to performance directly to make conclusions about compensation.

### Dedifferentiation and Aging

Dedifferentiation of visuospatial abilities was first demonstrated on behavioral tests. Young adults had very differentiated perceptual and spatial abilities (Chen et al., 2000), suggesting considerable functional benefits of performing different types of visual discrimination in a very specific manner. This specificity also had potential functional anatomic implications, such as the possibility that focal and spatial visual processing involved different cortical systems that acted somewhat independently (e.g., ventral vs. dorsal visual processing pathways). In contrast, a principal components analysis of these abilities in older adults showed only one common factor, suggesting dedifferentiation of those skills (Chen et al., 2002). This pattern of dedifferentiation was reflected in imaging studies. Research led by Grady et al. (1992) examined PET results during face and dot-location matching tasks in older and younger participants. While, in younger adults, face matching activated the occipitotemporal cortex and dot-location matching the superior parietal cortex, older adults had more occipitotemporal activation during location matching and more superior parietal activation during face matching. Similarly, Park et al. (2004) measured activation during encoding of different categories of stimuli (faces, places, non-sense words, and chairs) and showed that voxels that responded to one of the stimulus categories were more likely to respond to multiple categories in older compared to younger adults, demonstrating dedifferentiation in the older adults.

Age was unrelated to the extent of neural recruitment in the current study, a finding that differs from the findings of Cabeza (2001), Cabeza et al. (2002) While the reason for this discrepancy is not clear, several methodological differences exist between these past studies and the current investigation, which may account for a discrepancy in findings. First, prior research compared older and younger groups. Our participant sample did not include younger adults, so there was a more restricted age range. However, among middle-aged and older adults, age may be less determinant of neural recruitment than other factors. Examining age as a continuous variable in only middle-aged and older adults, as the current study did, may highlight differences associated with other variables, such as cognitive reserve, rather than age. Another clear difference is that other studies measured overlapping activation by indexing voxels that pass thresholds of significance. This can lead to problems when comparing activation between tasks or groups, as groups or tasks may have different activation intensities, which would lead to different significance thresholds and potential masking of effects (Zarahn et al., 2006; Voss et al., 2008).

The differentiation index employed in the current study was previously employed by Voss et al. (2008), who had adapted the method from studies of functional response specificity to faces in the inferior temporal/fusiform face area (Afraz et al., 2006; Grill-Spector et al., 2007). This method uses signal detection theory to calculate discriminability (d’), or the degree of selectivity among the three posterior ROIs. This measure considers the difference between ROI activation for the associated and unassociated tasks, normalized by the variability of activation in that ROI for all three tasks. By calculating d’, this and the Voss study had a quantitative measure of neural specificity to the preferred stimulus relative to the other tasks that could be compared to a continuous age measure. Like in prior research, the Voss et al. (2008) study examined differences between younger (aged 18–35 years) and older (aged 55–80 years) adults. Unlike the other studies, they examined differentiation indices as a function of age in just older adults as well, an approach that is consistent with the current study. Although older adults were less differentiated than younger adults in the Voss data, there was no effect of age when examining only older adults. This finding is consistent with the results of the current study and suggest that cortical dedifferentiation is not associated with age when only middle-aged and older adults are studied.

### Limitations and Future Directions

That there were not stronger age effects may reflect the restricted age range of this dataset, since no younger adults were included. Many studies show that older adults have more variability in functional activation than do younger adults (Li and Lindenberger, 1999; Ryan et al., 2012), and only when they are divided by performance do differences between older and younger adults emerge (Cabeza et al., 2002; Daselaar et al., 2003; Nyberg et al., 2003; Wingfield and Grossman, 2006). Given that younger adults were not included in the current study, and that age-related change in dedifferentiation could not be compared to performance directly, a conclusion regarding the compensatory effect of dedifferentiation could not be made. However, results add to a literature that suggests dedifferentiation could be compensatory, and future research would benefit from use of the differentiation index to compare the relationships between age, performance, and functional activation among younger and older adults.

While comparing an older to a younger group may be useful, the current focus on middle-aged and older adults suggests that there are not substantial age effects on differentiation. Future research should not only compare the current data with those from younger adults, but studies may also continue to examine performance effects among older adults, as variability in aging may cause differences in recruitment for those who perform well and those who perform poorly. As described, an ideal way to determine whether functional activation is compensatory is to directly compare performance to activity. However, regions activated may vary substantially based on experimental conditions. As such, it may be more comprehensive to characterize cognitive abilities by examining performance on a battery of cognitive tests, or to use a composite score to divide high and low performers (Daselaar and Cabeza, 2005). Future studies may attempt to not only compare functional activity during visual tasks to performance on those tasks, but also to performance on other types of visual tasks and to other cognitive performances.

## Conclusion

The current study provides evidence that greater dedifferentiation of activation across cortical areas involved in visual perception is associated with stronger visual discrimination performance, a finding that has implications with respect to both the functional significance of cortical dedifferentiation as well as age-associated changes in visual perception. That advanced age was not associated with major declines in discrimination accuracy, nor with differences in dedifferentiation, suggests that middle-aged and older adults are a heterogeneous group in which age becomes a less important predictor of changes in crystalized cognitive functions. Instead, performance and functional activation varied as a function of each other, with age as a non-significant variable. Our findings are consistent with prior research suggesting that primary visual perception is relatively stable as adults reach middle and older ages, though there are declines in processing speed. As such, other factors (e.g., cognitive reserve) may have more of an influence on functional activation and perceptual accuracy than age itself.

## Author’s Note

The content of this manuscript has previously appeared online as a thesis and can be found in the University of Florida digital collections of academic dissertations.

## Data Availability Statement

The raw data supporting the conclusions of this article will be made available by the authors, without undue reservation.

## Ethics Statement

The studies involving human participants were reviewed and approved by University of Florida Institutional Review Board. The patients/participants provided their written informed consent to participate in this study.

## Author Contributions

TS conducted the research it describes. RC, AW, and EP aided in the development of the study. EP and AW were integral in the implementation of the study, including designing the fMRI protocol. RC was instrumental in writing and editing the manuscript. All authors contributed to the article and approved the submitted version.

## Conflict of Interest

The authors declare that the research was conducted in the absence of any commercial or financial relationships that could be construed as a potential conflict of interest.

## Footnotes

1 http://www.fil.ion.ucl.ac.uk/spm
